# Supplementary material for: Real-space and real-time dynamics of CRISPR-Cas9 visualized by high-speed atomic force microscopy
Source: Nat Commun. 2017 Nov 10;8:1430. doi: 10.1038/s41467-017-01466-8 (PMC5681550; doi:10.1038/s41467-017-01466-8)
Supplement: Supplementary file 1 — Supplementary Information [file 41467_2017_1466_MOESM1_ESM.pdf]

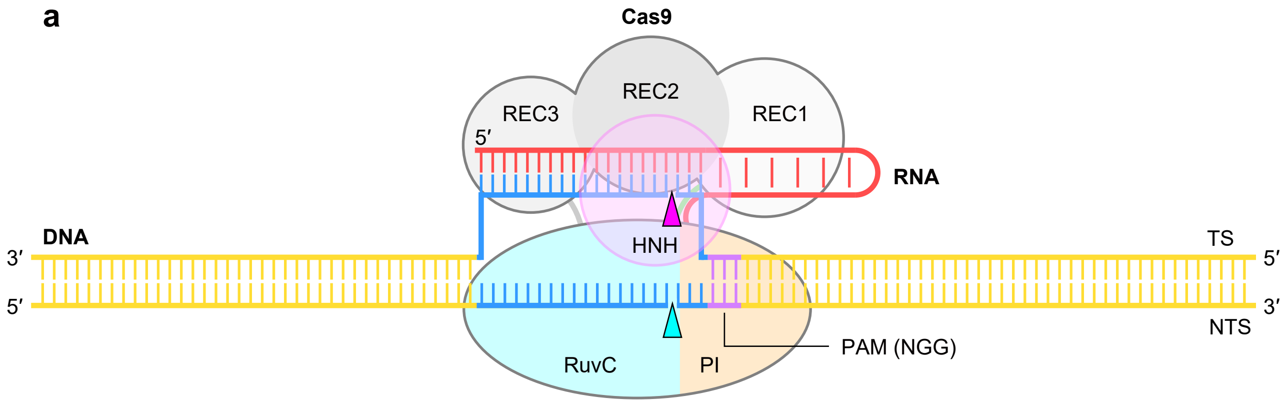

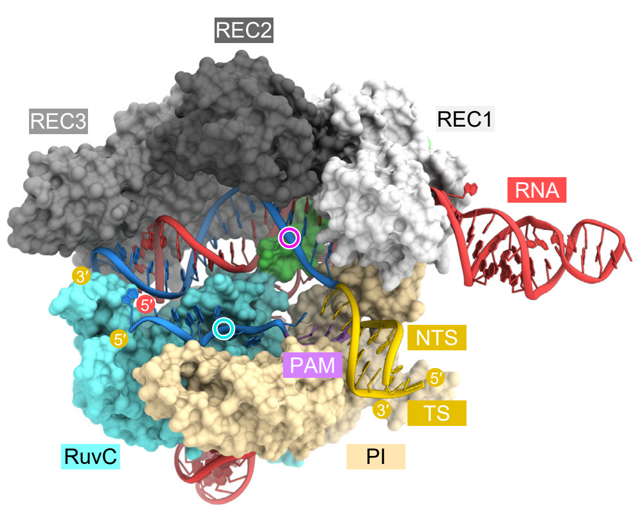

Supplementary Figure 1 | RNA-guided DNA cleavage by Cas9.
a Schematic of DNA cleavage by the Cas9–RNA complex. The 20-nucleotide guide segment in the crRNA hybridizes with the target DNA strand. The crRNA and the tracrRNA can be fused by an artificial linker to form a single-guide RNA. DNA cleavages by the RuvC and HNH domains are indicated by the cyan and magenta triangles, respectively. TS, target strand; NTS, non-target strand.
b Crystal structure of Cas9–RNA–DNA (PDB: 5F9R). The sites cleaved by the RuvC and HNH domains are indicated by the cyan and magenta circles, respectively. The HNH domain is omitted for clarity.

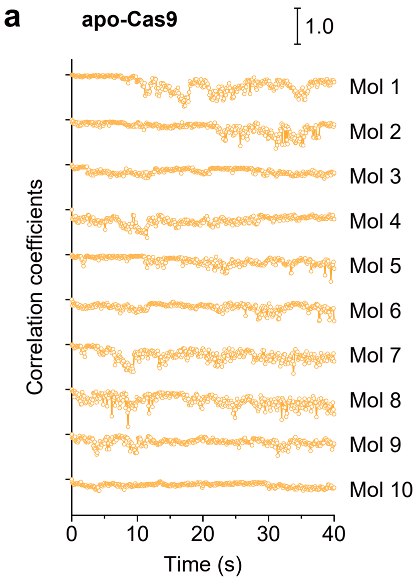

Supplementary Figure 2 | HS-AFM observations of apo-Cas9 and Cas9–RNA.
a, b Time courses of 2D correlation coefficients between the sequential HS-AFM images of ten representative apo-Cas9 (**a**) and Cas9–RNA (**b**) molecules.

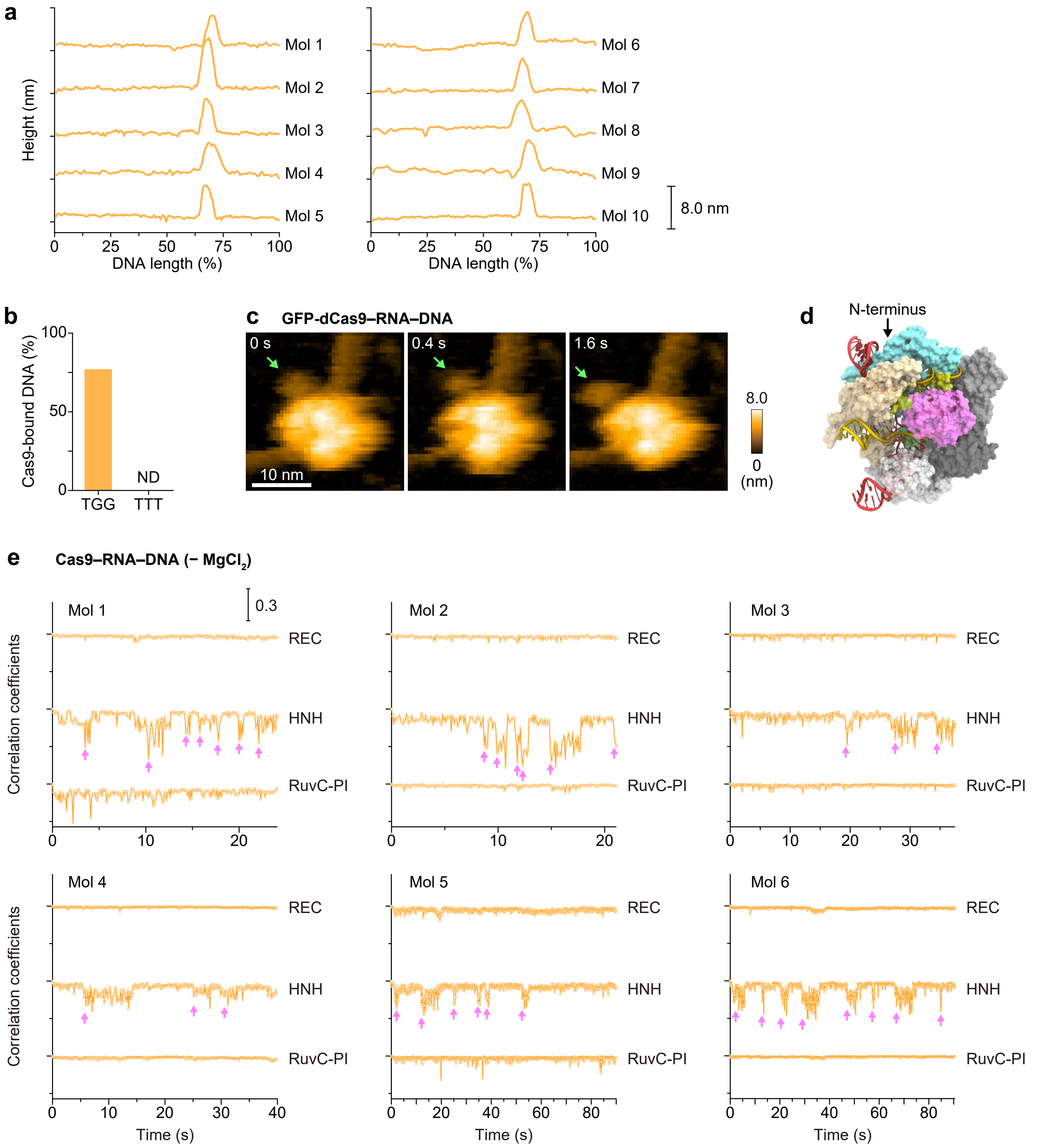

Supplementary Figure 3 | HS-AFM observations of Cas9–RNA–DNA.

a Cross-sectional profiles along DNA in ten representative HS-AFM images of Cas9–RNA–DNA. The lengths of the DNAs were normalized, since it is difficult to precisely measure their lengths in the HS-AFM images.

b Binding of Cas9–RNA to DNA with either the TGG PAM (n = 160) or the TTT PAM (n = 116). ND, not detected.

c Sequential HS-AFM images of GFP-dCas9–RNA–DNA. The GFP fused at the N-terminus of dCas9 is indicated by the green arrows.

d Location of the N-terminus of Cas9 (PDB: 5F9R). For comparison, the guide RNA was replaced with that in the Cas9–RNA–DNA structure (PDB: 4OO8), which is identical to that used for the HS-AFM observations.

e Time courses of 2D correlation coefficients for the individual domains between the sequential HS-AFM images of six representative Cas9–RNA–DNA molecules. The HNH domain fluctuations are indicated by magenta arrows.

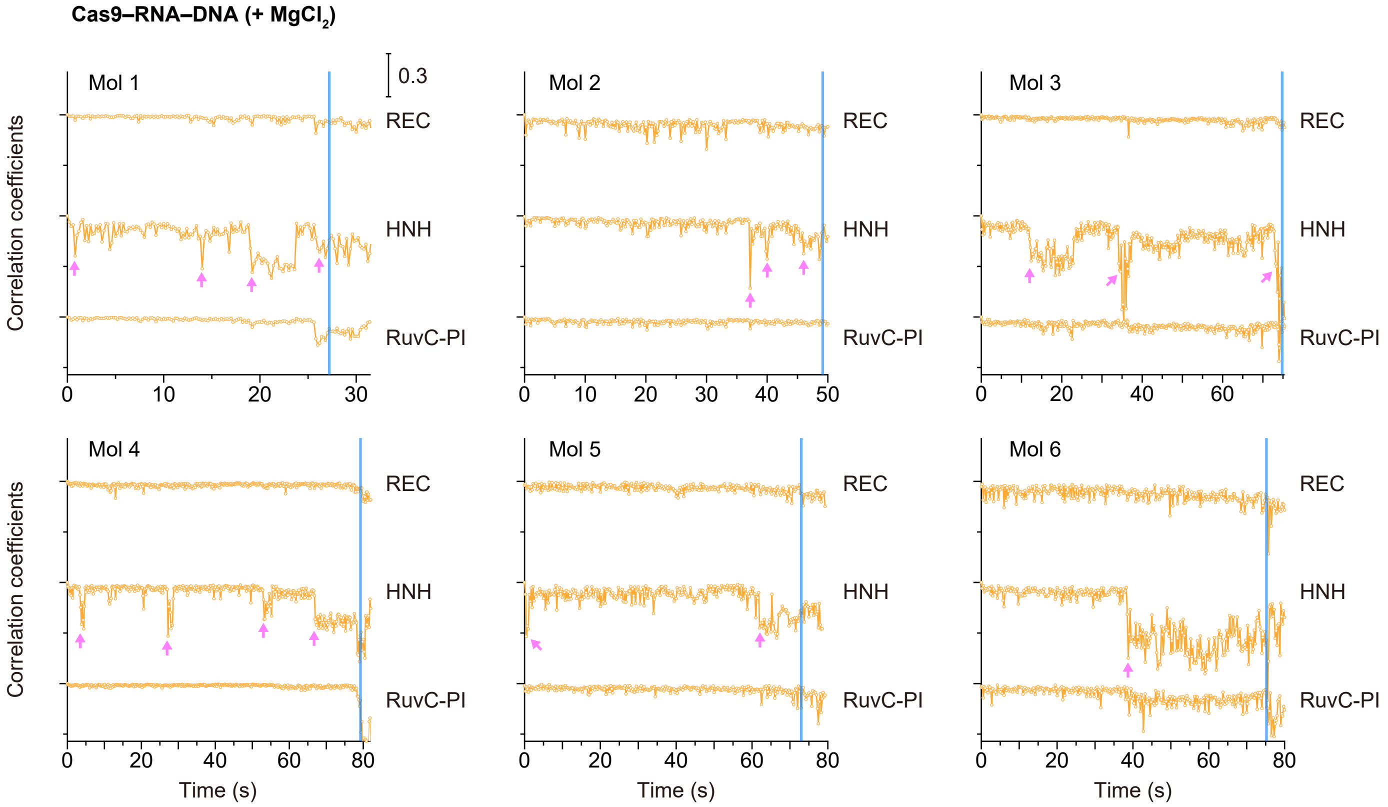

Supplementary Figure 4 | Conformational fluctuations of Cas9–RNA–DNA.
Time courses of 2D correlation coefficients for the individual domains between the sequential HS-AFM images are shown for six representative Cas9–RNA–DNA molecules in the presence of Mg^{2+}. The HNH domain fluctuations are indicated by magenta arrows. The cleavage product release is indicated by blue lines.

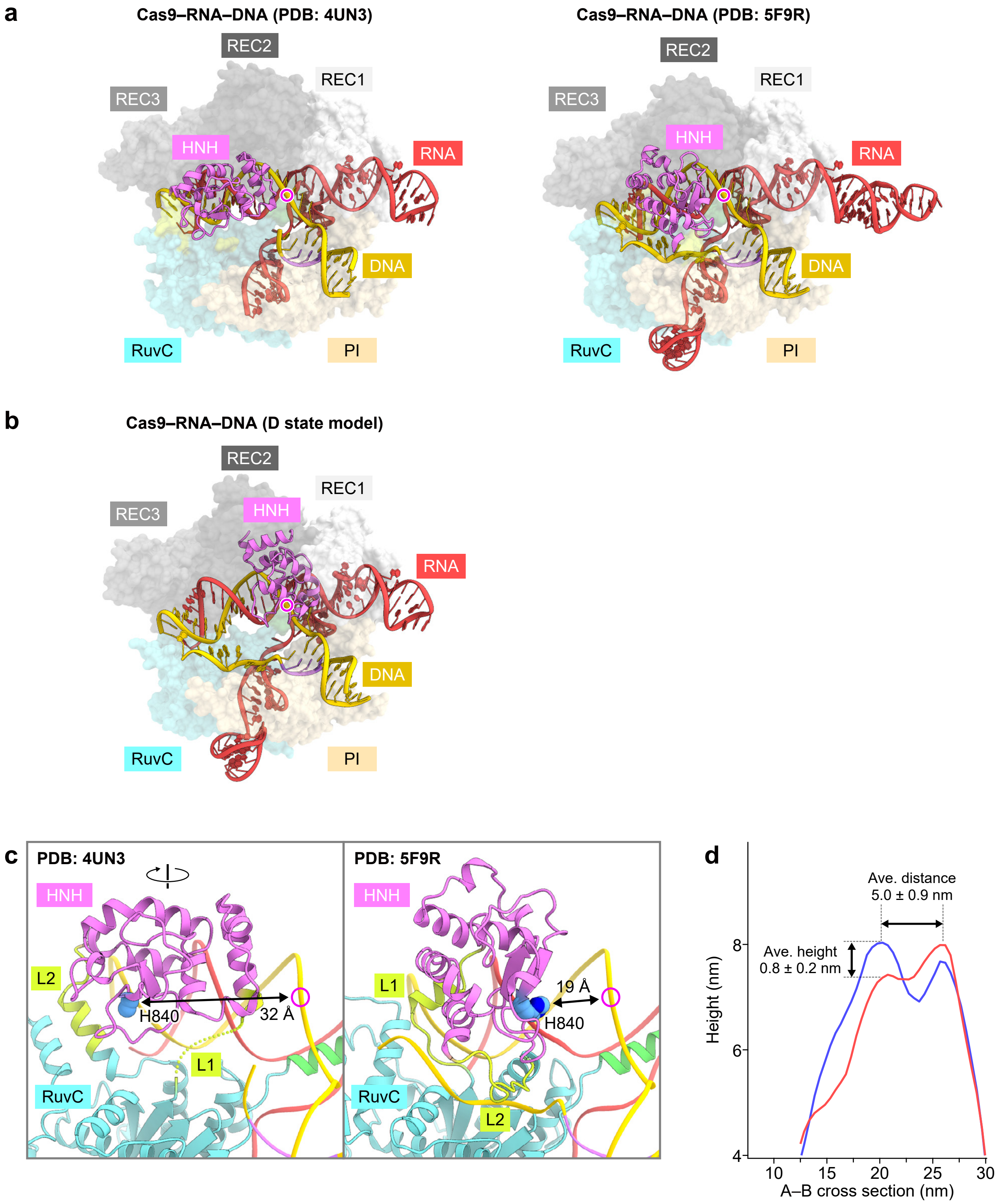

Supplementary Figure 5 | Structural rearrangement of the HNH domain for DNA cleavage.

a Crystal structures of Cas9–RNA–DNA (PDB: 4UN3 and 5F9R). The HNH cleavage sites are marked by magenta circles.

b Structural model of the catalytically-active D state of Cas9. The crystal structure of the EndoVII HNH nuclease bound to its target DNA (PDB: 2QNC) was superimposed onto Cas9–RNA–DNA (PDB: 4OO8), based on their target DNA strands. The Cas9 HNH domain was then superimposed onto the EndoVII HNH nuclease. The target DNA was replaced with that in the Cas9–RNA–DNA structure (PDB: 5F9R).

c Structural rearrangements of the L1 and L2 linkers in Cas9–RNA–DNA (PDB: 4UN3 and PDB: 5F9R). The HNH cleavage sites are marked by magenta circles.

d Cross-sectional profiles along the lines shown in Fig. 4 for representative Cas9–RNA–DNA molecules. The height distributions of the inactive and active states are indicated by blue and red lines, respectively (mean ± s.d., *n* = 14).

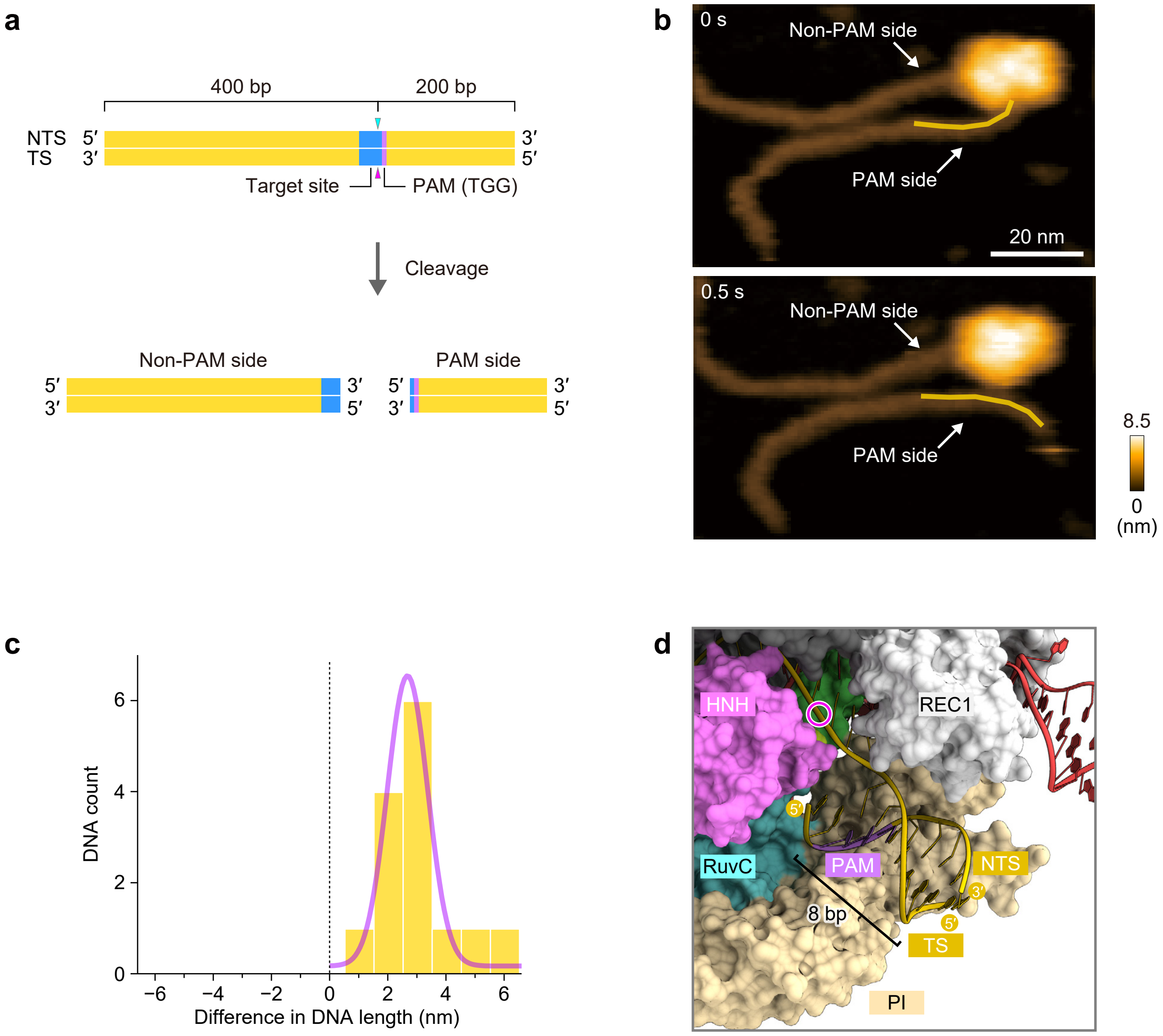

Supplementary Figure 6 | HS-AFM observations of the release of the cleavage products.
a Schematic of the target DNA cleavage. The target site and the PAM are colored blue and purple, respectively. The sites cleaved by the RuvC and HNH domains are indicated by the cyan and magenta triangles, respectively. TS, target strand; NTS, non-target strand.
b Sequential HS-AFM images of Cas9–RNA–DNA before and after the cleavage product release.
c Length differences in the PAM-side DNA before and after the release. The histogram fits a Gaussian curve, with the peak corresponding to 2.7 nm (n = 14).
d Binding of the PAM-containing DNA duplex to Cas9–RNA (PDB: 4UN3). The HNH cleavage site is indicated by a magenta circle. The 8-bp PAM DNA duplex is accommodated within the PAM-binding groove in the PI domain.

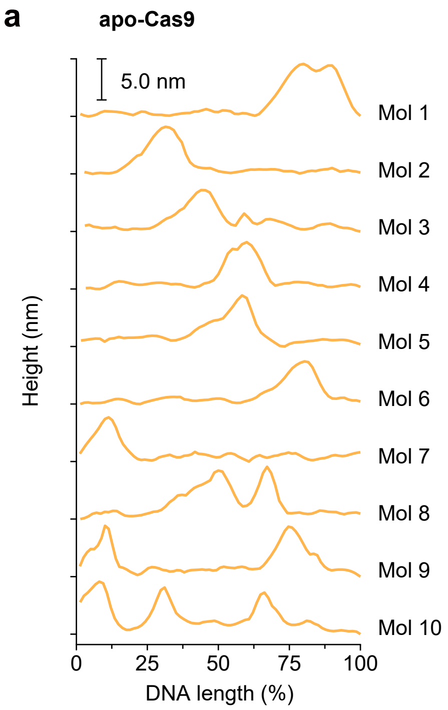

Supplementary Figure 7 | HS-AFM observations of the target search by Cas9–RNA.
a, b Cross-sectional profiles along the DNA in ten representative HS-AFM images of apo-Cas9 (**a**) and Cas9–RNA (**b**) on the lipid bilayer.
