# Supplementary material for: Real-space and real-time dynamics of CRISPR-Cas9 visualized by high-speed atomic force microscopy
Source: Nat Commun. 2017 Nov 10;8:1430. doi: 10.1038/s41467-017-01466-8 (PMC5681550; doi:10.1038/s41467-017-01466-8)
Supplement: Supplementary file 2 — Descriptions of Additional Supplementary Files [file 41467_2017_1466_MOESM2_ESM.pdf]

Description of Additional Supplementary Files

File Name: Supplementary Dataset 1
Description: Amino-acid and nucleotide sequences.

File Name: Supplementary Movie 1
Description: HS-AFM movies of three representative apo-Cas9 molecules on the AP-mica surface, showing that apo-Cas9 adopts flexible conformations. Pixel sizes: 1st movie, 48×45; 2nd movie, 50×48; 3rd movie, 54×48 pixels^2.

File Name: Supplementary Movie 2
Description: HS-AFM movies of three representative pre-assembled Cas9–RNA molecules on the AP-mica surface, showing that Cas9–RNA has a stable bilobed structure. Pixel sizes: 1st movie, 62×59; 2nd movie, 70×67; 3rd movie, 66×55 pixels^2.

File Name: Supplementary Movie 3
Description: HS-AFM movies of three representative pre-assembled Cas9–RNA–DNA molecules on the AP-mica surface, confirming that Cas9–RNA specifically binds to the target site. The HNH domain frequently disappeared and then reappeared at the original position. Fluctuations of the HNH domain are indicated by magenta arrows. Pixel sizes: 1st movie, 106×71; 2nd movie, 82×70; 3rd movie, 72×58 pixels^2.

File Name: Supplementary Movie 4
Description: HS-AFM movies of two representative pre-assembled GFP-dCas9–RNA–DNA molecules on the AP-mica surface, confirming the orientation of the Cas9–RNA molecule relative to the DNA. Fluctuations of the HNH domain are indicated by magenta arrows. GFP fused at the N-terminus of dCas9 is indicated by green arrows. Pixel sizes: 1st movie, 126×65; 2nd movie, 86×63 pixels^2.

File Name: Supplementary Movie 5
Description: HS-AFM movies of three representative pre-assembled Cas9–RNA–DNA molecules on the AP-mica surface, showing that the HNH domain undergoes fluctuations and then adopts the docked conformation, followed by the release of the cleaved DNA. The cleavage reaction was initiated by the addition of MgCl2 during the HS-AFM observations. Fluctuations of the HNH domain are indicated by magenta arrows. The cleavage products released from Cas9–RNA are indicated by blue arrows. Pixel sizes: 1st movie, 92×61; 2nd movie, 84×69; 3rd movie, 98×70 pixels^2.

File Name: Supplementary Movie 6
Description: Three representative HS-AFM movies of apo-Cas9 binding to the DNA on the lipid bilayer, showing that apo-Cas9 binds to the DNA in a non-specific manner. The target DNA was adsorbed onto the lipid bilayer, and apo-Cas9 was added during the HS-AFM observations. Apo-Cas9 molecules bound to the DNA are indicated by white arrows. Pixel size: 80×80 pixels^2.

File Name: Supplementary Movie 7
Description: Three representative HS-AFM movies of Cas9–RNA–DNA on the lipid bilayer, indicating that Cas9–RNA specifically binds to the target sites. The target DNA was adsorbed onto the lipid bilayer, and Cas9–RNA was added during the HS-AFM observations. Cas9–RNA molecules bound to the DNA are indicated by white arrows. Pixel size: 80×80 pixels^2.

File Name: Supplementary Movie 8
Description: HS-AFM movies of the DNA alone (left) and Cas9–RNA–DNA (right) on the lipid bilayer, indicating that Cas9–RNA searches for the target sites by three-dimensional diffusion. The target

DNA was adsorbed onto the lipid bilayer, and Cas9–RNA was added during the HS-AFM observations. Cas9–RNA molecules bound to the DNA are indicated by white arrows. Pixel size: 80×80 pixels^2.
